# Artificial Intelligence-Driven Nanobody Engineering for Pathogen Recognition: From Functional Epitope Prioritization to Multimodal Foundation Models

**DOI:** 10.3390/biom16101479

**Published:** 2026-10-11

**Authors:** Rui-Si Hu, Muhammad Ehsan, Wei Luo, Jian Zhou, Yong-Le Xiao, He-Xin Wang, Cheng-Yang Li, Kui Gu

**Affiliations:** 1 Innovation Team for Information Science and Biomedicine, School of Medicine, Sichuan University of Arts and Science, Dazhou 635000, China; grishu0707@gmail.com (R.-S.H.); ww_luo@sasu.edu.cn (W.L.); zj2020@sasu.edu.cn (J.Z.); yonixiao@sasu.edu.cn (Y.-L.X.); wanghexin.bio@gmail.com (H.-X.W.); chengyang.li2@sasu.edu.cn (C.-Y.L.); 2 Department of Parasitology, Faculty of Veterinary and Animal Sciences, The Islamia University of Bahawalpur, Bahawalpur 63100, Pakistan; 3 School of Pharmacy and Bioengineering, Chongqing University of Technology, Chongqing 400054, China

**Keywords:** nanobody, artificial intelligence, pathogen recognition, functional epitope prioritization, de novo design, multimodal learning

## Abstract

Nanobodies are compact and stable proteins derived from the variable domain of camelid heavy-chain-only antibodies (VHH). Advances in structural biology, molecular modeling, and artificial intelligence (AI) are shifting VHH discovery from empirical selection toward data-driven design. However, predicted binding alone does not ensure biological activity or successful translation, particularly for antigenically variable pathogens. Complementing recent reviews that broadly survey computational VHH modeling and optimization, this review focuses on how antigen biology and functional epitope prioritization can guide the design of pathogen-targeting VHHs. We organize the field along a structure–function–translation continuum, connecting the structural basis of VHH recognition and available data resources with conventional discovery, antigen-guided de novo design, and sequence–structure learning. We further examine how developability, recombinant production, multispecificity, humanization, structural dynamics, and antigenic variation shape the progression from predicted binders to functional VHHs. Finally, we discuss how multimodal foundation models could integrate these dimensions to support the design of functional, manufacturable, and escape-resilient VHHs.

## 1. Introduction

Nanobodies are single-domain antigen-binding fragments derived from the variable domain of camelid heavy-chain-only antibodies (VHH), first identified in the early 1990s [1]. With a molecular weight of approximately 15 kDa, VHHs combine compact size with high solubility, physicochemical stability, and efficient recombinant production [2,3]. Their often-extended complementarity-determining region 3 (CDR3) loops can access grooves, cavities, and recessed epitopes that are less accessible to conventional antibodies (Ab) [3,4,5]. VHHs can also be engineered into multivalent or bispecific formats, fusion proteins, and diagnostic probes for therapeutic, diagnostic, and research applications [6,7]. They are particularly valuable for pathogen recognition, where biological activity depends not only on binding affinity but also on the location and functional relevance of the recognized epitope [8].

Pathogen antigens are dynamic targets. Mutation, recombination, glycan shielding, conformational changes, and stage-specific expression can alter antigen availability, epitope exposure, and Ab recognition [9,10]. Consequently, high affinity alone does not guarantee neutralization or functional inhibition. VHHs targeting functionally irrelevant epitopes may bind strongly without affecting pathogen entry, replication, virulence, or toxin activity. Rational VHH design should consider epitope accessibility, structural conservation, and biological function, particularly for regions involved in receptor binding, membrane fusion, enzymatic activity, or toxin action [11,12,13]. Distinguishing molecular binding from functional activity is thus central to pathogen-targeting VHH design.

Conventional VHH discovery commonly uses immune, naïve, or synthetic libraries followed by iterative display-based selection [14]. Although effective, these workflows could be laborious and costly. Advances in structural biology and AI are expanding VHH engineering beyond empirical selection. Recent developments include protein language models (PLMs) that learn contextual sequence representations for mutation-effect and protein-property prediction [15,16], geometry-aware and graph-based models that capture spatial residue organization and antigen-binding interfaces [17,18,19], and generative models that enable CDR design, backbone generation, and antigen-conditioned binder design [20,21]. Together with growing Ab repertoires, structural databases, and VHH–antigen complex data, these developments are moving the field toward integrated sequence–structure and multimodal modeling.

Recent reviews [22,23,24,25] have covered conventional and AI-assisted VHH discovery, computational modeling, multiepitope engineering, and humanization (Table 1). Here, we take a pathogen-focused view and ask how epitope choice can direct computationally identified binders toward biologically meaningful outcomes. We trace VHH development from recognition principles and data resources to library-based discovery, antigen-guided de novo design, and sequence–structure learning. We also examine how antigenic variation and translational factors, including developability, production, multispecificity, humanization, and structural dynamics, shape candidate progression. Collectively, these topics provide a structure–function–translation perspective on the development of functional pathogen-targeting VHHs.

## 2. Structural and Functional Foundations of VHHs

### 2.1. Structural Features Underlying VHH Recognition

**Figure 1:**
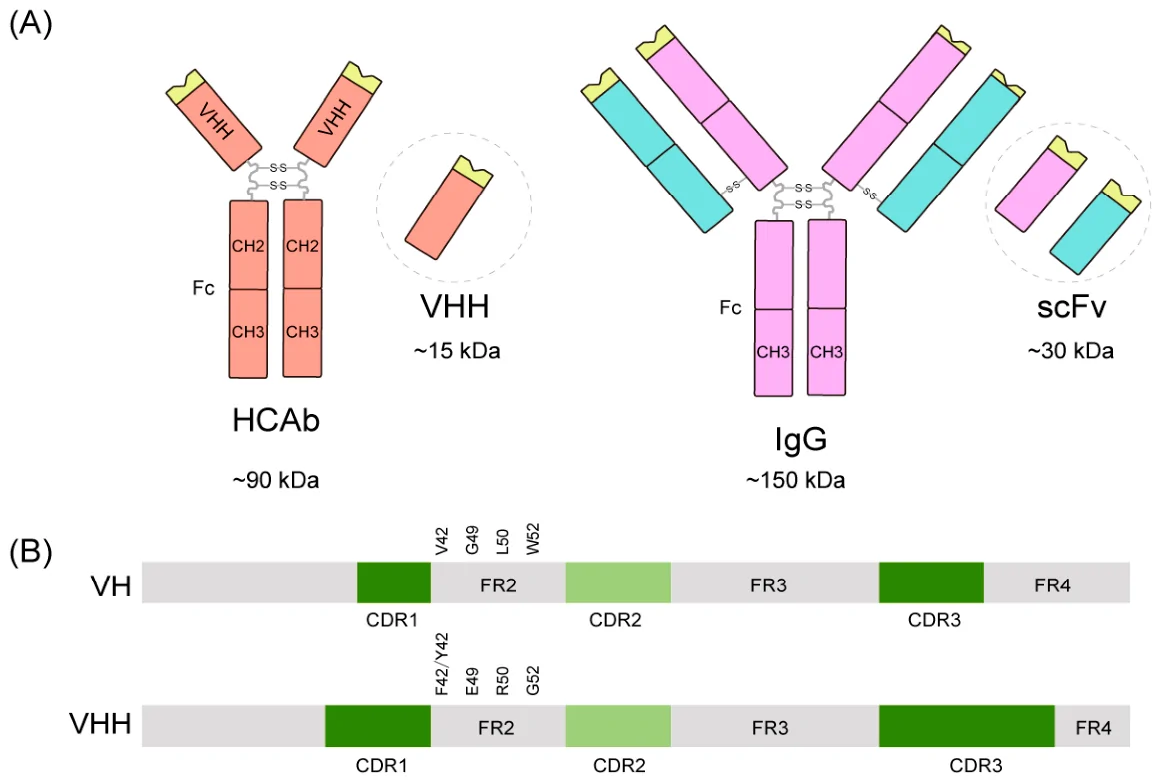
Structural features of VHHs compared with conventional Abs. (**A**) VHHs are ~15-kDa variable domains derived from camelid HCAbs (~90 kDa) and are substantially smaller than conventional IgG Abs (~150 kDa) and single-chain fragment variable (scFv) (~30 kDa). (**B**) VH and VHH domains share three CDRs and four framework regions, whereas VHHs contain characteristic FR2 substitutions that reduce the hydrophobicity of the former VH–VL interface and often possess a longer and structurally diverse CDR3. Residues are numbered according to the IMGT scheme.

VHHs are the antigen-binding variable domains of camelid heavy-chain-only antibodies (HCAb; ~90 kDa) and their isolated single-domain binders have a molecular mass of approximately 15 kDa, substantially smaller than conventional IgG Abs (~150 kDa) and scFv (~30 kDa) (Figure 1A) [26]. Like conventional VH domains, VHHs contain four framework regions (FR1–FR4) and three complementarity-determining regions (CDR1–CDR3). However, VHHs differ in several framework residues that normally contribute to the VH–VL interface and often possess a longer and more structurally diverse CDR3 (Figure 1B) [27,28]. Characteristic substitutions at IMGT positions 42, 49, 50 and 52 reduce surface hydrophobicity and contribute to VHH solubility [29,30]. Moreover, many VHHs contain an extended CDR3 loop that is often stabilized by an additional disulfide bond. Beyond antigen binding, these extended loops may also contribute to VHH solubility.

### 2.2. Structural Basis of VHH–Pathogen Recognition

The compact VHH scaffold and flexible CDR loops, especially the extended CDR3, enable access to the recessed or sterically restricted epitopes [28]. Early VHH–lysozyme structures showed that a CDR3 loop can penetrate enzyme active-site clefts [5]. Direct comparisons with conventional Abs further demonstrated the preferential targeting of antigen clefts by VHHs [31]. These features support recognition of receptor-binding interfaces and other functional pathogen sites. However, biological activity depends not only on binding affinity but also on epitope accessibility, binding orientation, conservation and function [7,32,33]. Conserved and functionally constrained epitopes may therefore be better targets for disrupting essential pathogen processes, providing a basis for functional epitope prioritization in computational design.

## 3. Databases and Data Resources for VHH Development

Sequence, structural and functional information provides the foundation for VHH discovery. The rapid accumulation of experimentally characterized and computationally generated VHH data has led to the establishment of specialized databases that support sequence analysis and computational design. As summarized in Table 2, these resources provide different levels of information, including VHH sequences, three-dimensional (3D) structures, antigen specificity, binding affinity and biophysical properties. Most databases provide downloadable data in standardized formats (e.g., FASTA, CSV and PDB), while several platforms additionally integrate analytical tools for sequence search, structural comparison, docking and AI-assisted prediction.

### 3.1. Sequence Databases

Sequence databases represent the largest data source for VHH analysis and model training. The application of next-generation sequencing and synthetic library technologies has substantially expanded experimentally validated and engineered sdAb repertoires. The sdAb-DB [42] contains more than 1400 manually curated sdAb sequences collected from literature and public repositories, providing a resource for sequence retrieval and diversity analysis. Recently, PLAbDab-nano [43] has expanded the available repertoire by integrating nearly 5000 VHH, VNAR and other sdAb sequences. The database supports flexible searches based on keywords, regular expressions and metadata annotations, facilitating repertoire exploration, sequence comparison and Ab engineering.

### 3.2. Structure Databases

Structural databases provide essential resources for understanding VHH folding, antigen recognition and molecular interactions. The PDB [47] remains the primary repository for experimentally resolved macromolecular structures and contains an increasing number of VHH and VHH–antigen complex structures. Based on PDB entries, SAbDab-nano [44], a dedicated subset of SAbDab2 [45], systematically collects experimentally determined sdAb structures, including camelid VHH and shark VNAR. Each entry is annotated with structural coordinates, experimental methods, resolution, antigen information, chain classification and 3D visualization. In addition, SNAC-DB [46] provides curated Ab–antigen and VHH–antigen complex structures designed for structural benchmarking, docking studies and machine learning applications.

### 3.3. Functional and Application-Oriented Databases

Beyond sequence and structural resources, several databases incorporate experimentally measured functional properties relevant to VHH engineering. NanobodyThermo [40] compiles more than 560 manually curated VHHs with experimentally determined melting temperatures, sequences and associated annotations, providing a resource for thermostability prediction and protein optimization. Thera-SAbDab [38] links therapeutic Abs and sdAb-derived therapeutics with available sequence and structural information. The Antibody and Nanobody Design Dataset (ANDD) [41] integrates sequence, structure, antigen information and binding affinity data from multiple sources, providing a benchmark dataset for deep learning (DL)-based Ab and VHH design. Disease-focused databases such as CoV-AbDab [39] contain experimentally characterized Abs and VHHs targeting coronaviruses, including SARS-CoV-2, SARS-CoV-1 and MERS-CoV, enabling pathogen-specific repertoire analysis.

Several integrated platforms, including INDI2 [34], NanoLAS [35,36] and iCAN [37], further combine sequence, structural and functional information with online analytical tools, supporting comprehensive VHH characterization and computational analysis.

## 4. Conventional VHH Discovery and Selection

**Figure 2:**
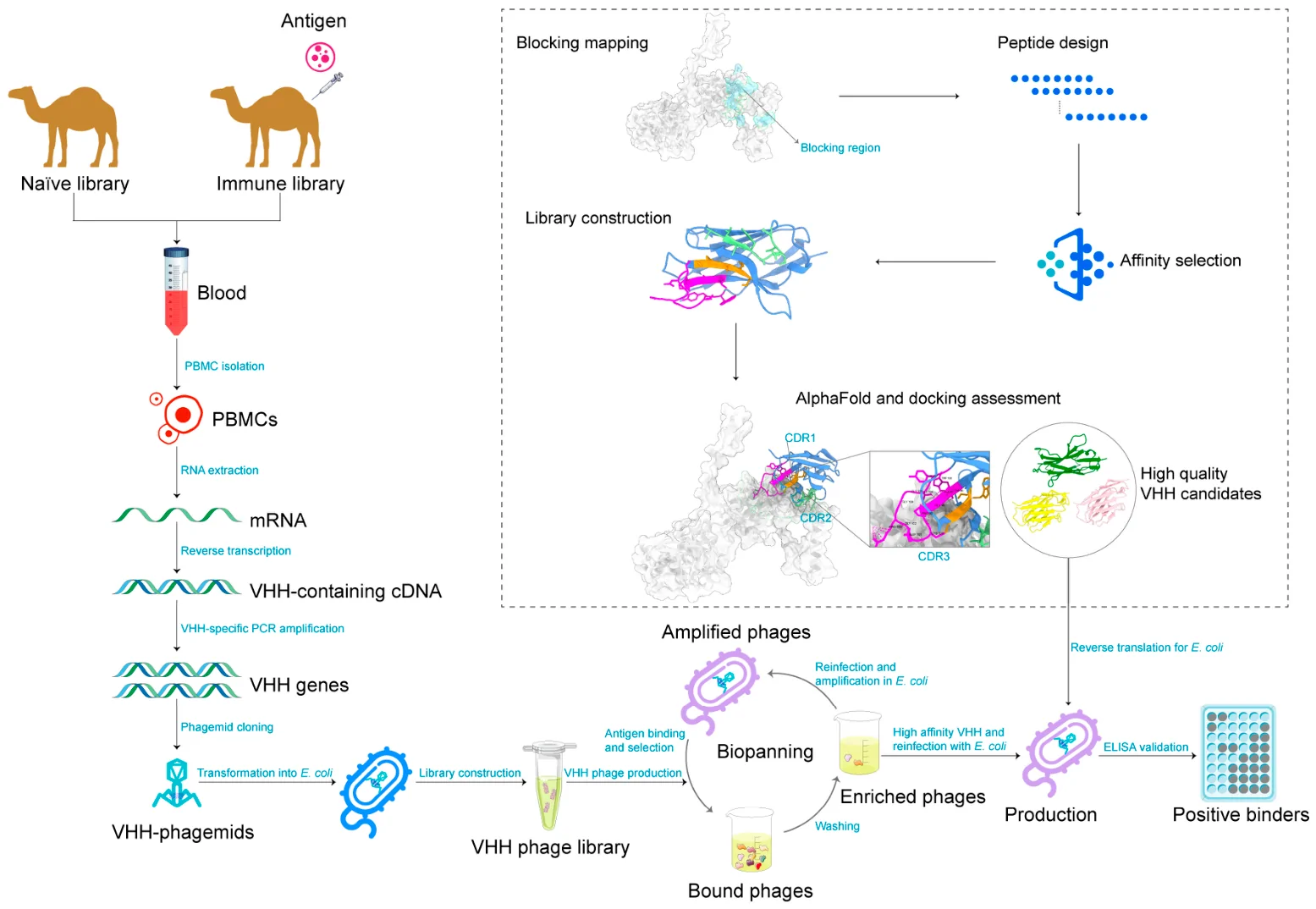
Conventional and computational workflows for VHH discovery and design. The left panel illustrates conventional VHH discovery using camelid-derived immune or naïve libraries. Following blood collection, PBMC isolation, mRNA extraction, reverse transcription, and VHH amplification, phage display libraries are constructed. Antigen-specific binders are then enriched through iterative biopanning and validated experimentally. The right panel illustrates an antigen-guided computational design workflow in which functional epitopes or design hotspots are first identified, followed by backbone or CDR generation, sequence design and optimization, complex prediction, and interface-based filtering. Candidate VHH sequences are then prioritized for synthesis and experimental validation.

Conventional VHH discovery mainly relies on camelid-derived naïve or immune VHH libraries combined with display-based selection (Figure 2). Naïve libraries are generated from non-immunized animals and provide antigen-independent sequence diversity, often reaching 10^9^–10^11^ variants, although selected binders may require subsequent affinity maturation [48,49]. In contrast, immune libraries are generated following antigen immunization and benefit from in vivo affinity maturation. They often yield antigen-specific VHHs with high affinity and specificity, but their construction depends on animal immunization and antigen availability [50,51].

In a typical immune-library workflow, VHH genes are amplified from cDNA prepared peripheral blood lymphocytes (PBMCs), cloned into display vectors, and subjected to iterative antigen selection, followed by validation of positive clones [7,32,33]. Although conventional approaches remain highly effective, candidate discovery depends strongly on library quality and experimental selection and provides limited control over epitope specificity before screening. These limitations motivate structure- and function-guided strategies in which target antigen regions are defined before VHH candidate generation.

## 5. Function-Guided Computational Design of Pathogen-Targeting VHHs

Unlike conventional discovery strategies, which commonly rely on library selection and often involve animal immunization, de novo computational VHH design aims to generate candidates directly from antigen structural information and predefined functional epitopes. Suitable antigen regions are first identified, followed by structure-guided design, sequence optimization, and computational evaluation to prioritize VHH candidates with favorable predicted binding properties.

However, the computational methods used across this workflow were developed for corresponding molecular systems and are supported by different levels of VHH evidence. We therefore group them into four categories: (i) VHH-focused methods evaluated on VHH data; (ii) methods evaluated on both conventional Abs and VHHs; (iii) Ab-focused methods with plausible but limited VHH evidence; and (iv) general protein-design methods used within VHH workflows. For the first two groups, we indicate whether the VHH evidence is computational or experimental. This distinction matters because VHHs lack a paired VL domain and differ from conventional Abs in framework architecture, CDR organization, antigen-contact patterns, and CDR3 geometry. Methods developed for conventional Abs or general proteins are therefore treated as candidates for adaptation rather than established VHH methods when VHH-specific evidence is lacking.

### 5.1. Application-Guided Functional Epitope and Hotspot Prioritization

The first step in de novo VHH design is to identify potentially targetable regions of a given antigen. Candidate regions are commonly evaluated on the basis of antigen function, structural context, sequence conservation, surface exposure, and Ab accessibility [52,53], but the relative importance of these criteria depends on the intended application. For diagnostic VHHs, the target region should support sensitive and specific detection and is therefore selected according to antigen abundance, assay accessibility, stability, and distinction from related organisms. For direct neutralization, priority is given to epitopes involved in receptor engagement, membrane fusion, or other essential steps in infection. Enzyme-inhibiting and toxin-blocking VHHs should target catalytic, substrate-binding, allosteric, receptor-binding, translocation, or oligomerization sites whose engagement is expected to alter biological activity. Pathogen biology provides the corresponding search space, e.g., viral targets commonly include entry proteins, nucleoproteins, and replication enzymes [54]; bacterial targets include adhesins, toxins, surface-associated virulence factors [55]; and parasite targets include invasion ligands, stage-specific surface antigens, and secreted enzymes [56]. Table 3 summarizes the antigen classes and regions commonly considered for computational target selection across pathogen types. Experimentally evaluated VHH targets are included only as examples of these antigen classes.

The functional regions highlighted in Table 3 are particularly relevant to direct neutralization or blockade, but protection in vivo can also arise through other immune mechanisms. For example, although unmodified VHHs lack an Fc domain, engineered formats can recruit Fc-dependent effector functions, including Ab-dependent cellular cytotoxicity (ADCC) and opsonophagocytosis, through Fc fusion or indirect engagement of endogenous immunoglobulins [80,81]. Multivalent VHHs may also protect through pathogen cross-linking and agglutination, as shown for F4-positive enterotoxigenic *E. coli* [66]. Thus, epitope selection should prioritize functional-site overlap for direct blockade, whereas accessibility, antigen density, and spatial arrangement may be more important for effector recruitment or agglutination.

Once candidate antigen regions have been selected according to the intended mechanism, computational analyses can refine them into functionally relevant and structurally suitable design hotspots (Table 4). Functional annotation first provides information on antigen topology and biological relevance by identifying signal peptides, transmembrane regions, conserved domains, and functional motifs [82,83,84]. Structural information is then used to determine whether candidate regions are accessible in the antigen state relevant to the intended application. Experimental structures in the Protein Data Bank (PDB) [85] provide templates for analyzing known ligand-, receptor-, or Ab-binding interfaces. When experimental structures are unavailable, AlphaFold-based methods can provide antigen or complex models for further structural evaluation [86,87]. However, predicted complexes are not equivalent to experimentally resolved Ab–antigen or VHH–antigen structures. Even when individual chains are modeled confidently, uncertainty in their relative orientation and interface geometry can lead to incorrect epitope placement. Complex predictions should therefore be assessed using local, pairwise, and interface-level confidence metrics, such as predicted aligned error (PAE) and interface predicted Template Modeling score (ipTM). These models can support interface prioritization but cannot establish binding or function without experimental validation.

Oligomerization, glycan shielding, and conformational changes should be considered in accessibility assessment, because epitopes on pathogen surface glycoproteins may be less accessible than suggested by static structures. Surface accessibility, electrostatic potential, and local physicochemical properties can further identify patches, grooves, and other regions suitable for VHH recognition [88,89,90]. For pathogen proteins involved in host receptor binding or protein–protein interactions, interface analysis can identify functionally important surfaces for blockade. A conserved but inaccessible region should generally be deprioritized unless it becomes exposed in a targetable state. Conversely, an accessible but variable region may be suitable for strain-specific detection but may be less suitable for broad or escape-resistant applications. Conservation analysis can identify regions with limited sequence variation, especially in highly variable viral and parasite antigens [91]. However, sequence conservation alone does not always reflect evolutionary or functional constraint. Some conserved regions may tolerate mutations, whereas functionally important regions may be constrained because escape mutations disrupt protein stability, receptor interactions, catalytic activity, or pathogen fitness. Therefore, escape-resistant epitope prioritization should consider both sequence conservation and the potential consequences of escape mutations, including their effects on pathogen fitness and their biological function. B-cell epitope prediction tools may provide complementary information on Ab-accessible regions [92,93,94,95], but should be interpreted cautiously because sequence-based predictions do not fully capture conformational epitopes [96]. By integrating functional annotation, structural accessibility, evolutionary evidence, and interface information, broad antigen regions can be narrowed into exposed and biologically relevant hotspots for downstream de novo VHH generation.

**Table 4:** Representative computational resources supporting functional epitope and hotspot prioritization.

Analytical Task	Purpose in De Novo VHH Design	Representative Tools or Databases
Functional annotation	Defines antigen regions, including signal peptides, transmembrane regions, domains, motifs, and post-translational modification (PTM) sites	SignalP [83]; DeepTMHMM [82]; Phobius [97]; DeepMVP [98]; UniProtKB/Swiss-Prot [99]; InterPro [84]
Experimental structure retrieval	Provides experimentally supported antigen structures and known receptor-, ligand-, or Ab-binding interfaces	SAbDab [44]; RCSB PDB [85]; PDBe [100]
Antigen structure prediction	Generates antigen structures when experimental models are unavailable and provides structural confidence metrics	AlphaFold DB [101]; AlphaFold 3 [87]; ColabFold [102]; RoseTTAFold [103]
Antigen–receptor interface modeling	Identifies potential receptor-binding regions targeted by competitive VHHs	AlphaFold 3 [87]; HDOCK [104]; HADDOCK [105]; ClusPro [106]
Surface accessibility analysis	Identifies solvent-exposed residues that are accessible to VHH CDR loops	FreeSASA [107]; DSSP [108]; ChimeraX [109]
Electrostatic and surface-property analysis	Detects charged patches, grooves, or physicochemical features favorable for CDR-mediated binding	ChimeraX [88]; PyMOL [110]
Conservation analysis	Prioritizes conserved surface regions for broad-spectrum or escape-resistant design	BLAST [111]; MAFFT [112]; ConSurf [113]
B-cell epitope prediction	Provides auxiliary evidence for Ab-accessible antigenic surfaces	BepiPred [114]; DiscoTope [92]

### 5.2. Structure-Guided Generative VHH Design

Once functional antigen regions and design hotspots have been defined, structure-guided generative approaches can be applied to generate VHH candidates against predefined epitopes. Unlike conventional docking-based strategies that mainly evaluate existing scaffolds, generative design approaches aim to jointly optimize antigen–VHH geometry, backbone conformations, and sequence compatibility. However, because computational generation can produce diverse candidate structures, reliable prioritization criteria are essential for identifying designs with biologically meaningful antigen recognition.

**Figure 3:**
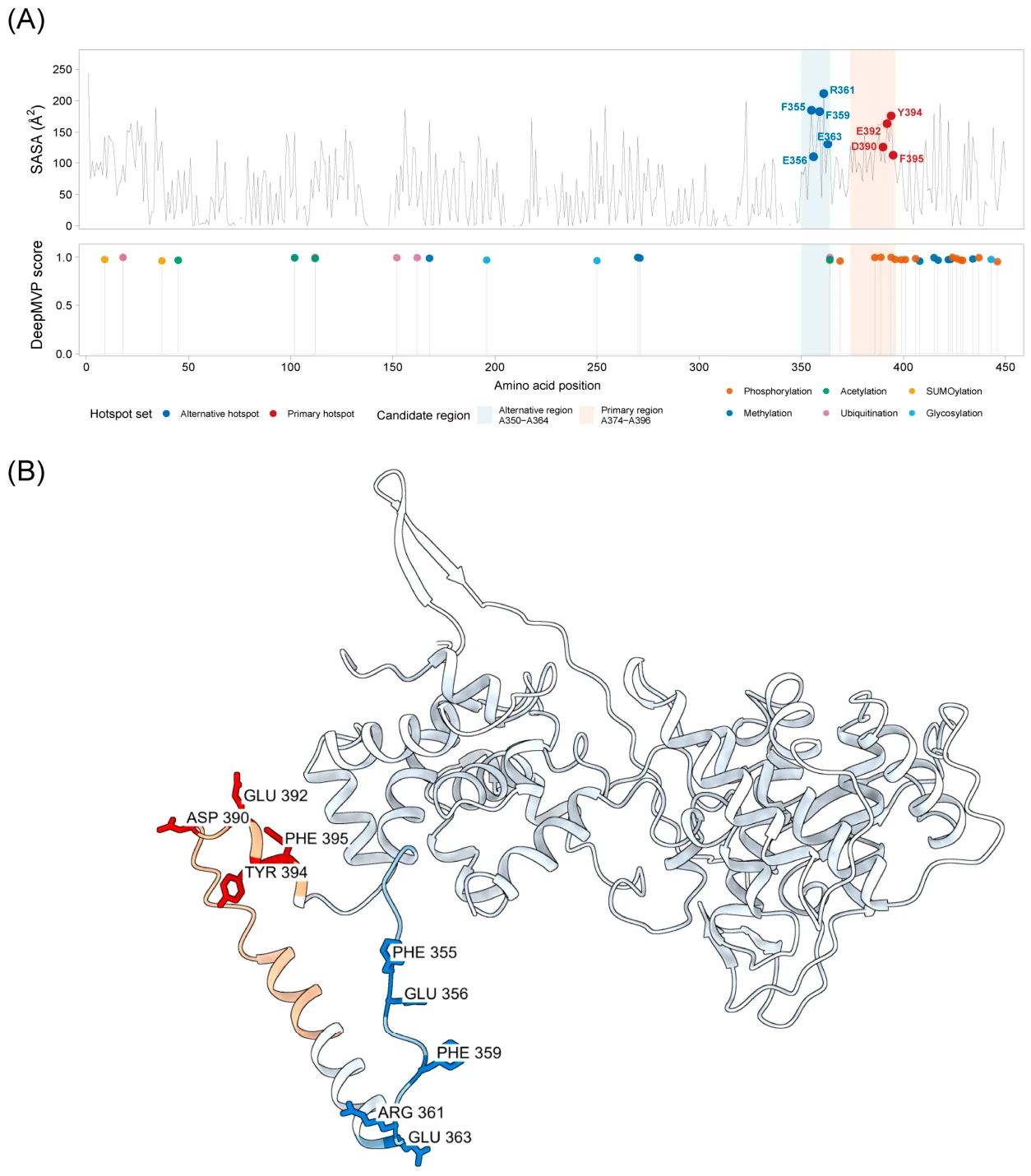
Illustrative computational hotspot selection based on surface accessibility in rabies virus nucleoprotein (GenBank accession: NP_056793.1). (**A**) Candidate regions were evaluated using residue-level SASA together with DL-based PTM-related annotations. Residues with SASA >100 Å^2^ were prioritized as highly exposed, whereas PTM predictions were used only as auxiliary residue-level information. These signals were mainly enriched in the N-terminal region and residues 350–450. (**B**) AlphaFold 3-based structural mapping of candidate residues showing their surface accessibility and spatial distribution for subsequent VHH design. It should be noted that the selected residues represent only illustrative, exposure-based computational hotspots and have not been experimentally validated to constitute a functional epitope.

An illustrative hotspot-selection process is presented using rabies virus nucleoprotein as an example (Figure 3). Antigen topology and surface properties were first evaluated for computational design. SignalP [83] and DeepTMHMM [82] predicted no signal peptide or transmembrane helices, consistent with the intracellular localization of the nucleoprotein. DeepMVP [98] identified potential PTM-associated residues in the C-terminal region and was used only as auxiliary annotation. Solvent-accessible surface area (SASA) analysis using ChimeraX [109] identified an exposed surface patch spanning residues 374–396 (Figure 3A). Based primarily on surface exposure and local structural context, Asp390, Glu392, Tyr394 and Phe395 were selected as illustrative design hotspots, while residues 350–364 were retained as an alternative exposed region (Figure 3B). However, these residues were selected solely through computational screening and require further experimental validation of their accessibility and functional relevance.

**Figure 4:**
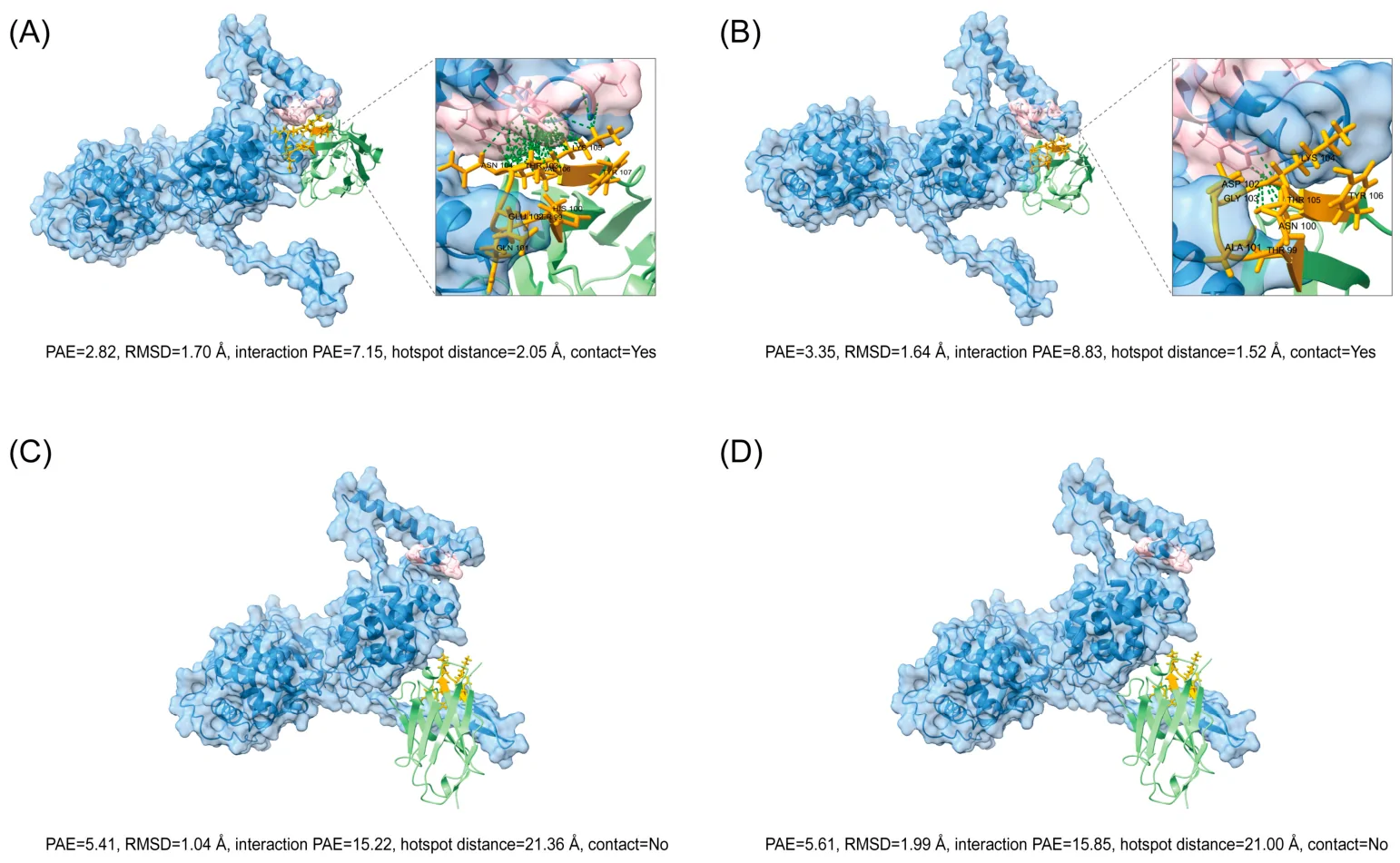
Illustrative RFantibody-based computational design against rabies virus nucleoprotein. The exposure-based hotspot residues Asp390, Glu392, Tyr394 and Phe395 were specified as design constraints. RFantibody was run with 500 backbone designs, CDR loop lengths of H1:7, H2:6 and H3:5–13, five sequences per backbone, a ProteinMPNN sampling temperature of 0.1 and 10 RF2 recycling iterations. Candidates were ranked using RF2 PAE, backbone RMSD, interaction PAE, hotspot distance and direct hotspot contact. Minimal heuristic filters included RF2 PAE < 10 and RMSD < 2 Å, with Rosetta ΔΔG < −20 used as an optional criterion. Panels (**A**,**B**) show predicted complexes with direct CDR3–hotspot contact, whereas panels (**C**,**D**) show designs that passed the numerical filters but lacked direct CDR3 contact with the selected hotspots. It should be noted that all designs are untested computational candidates and that predicted contacts and confidence metrics do not demonstrate antigen binding or functional activity.

Using these illustrative hotspot residues as design constraints, RFantibody [21] was applied to demonstrate an epitope-conditioned computational design workflow (Figure 4). The workflow combines an Ab-specialized RFdiffusion model [115] for backbone generation, ProteinMPNN [116] for sequence design, and an Ab-adapted RoseTTAFold2 [21] for complex prediction and filtering. The resulting designs are computational candidates and should not be interpreted as validated binders.

Candidate ranking considered predicted aligned error (PAE), backbone root-mean-square deviation (RMSD), interaction PAE, hotspot-to-CDR distance, and direct residue contact. In the rabies virus nucleoprotein example, the designs in Figure 4A,B showed predicted CDR3–hotspot contacts and therefore agreed more closely with the specified design constraint. In contrast, the designs in Figure 4C,D passed the numerical filters but lacked direct CDR3 contact with the selected hotspots. These computational metrics support candidate ranking but do not demonstrate binding or biological activity. Experimental validation is therefore required.

### 5.3. Scaffold-Based CDR Redesign and Candidate Refinement

In contrast to antigen-conditioned approaches that generate new VHH backbones from antigen structures, scaffold-based strategies start from predefined VHH frameworks or existing VHH candidates and mainly optimize antigen-contacting regions, particularly the CDR loops. Because CDRs, especially CDR3, contribute substantially to antigen recognition while remaining conformationally flexible, their redesign requires careful consideration of CDR conformation, VHH–antigen interface geometry, and framework stability [117,118]. This strategy is useful for improving existing VHH candidates or generating alternative CDR conformations while retaining the overall VHH framework.

NanoDesigner [117] is a VHH-oriented framework that integrates structure prediction, VHH–antigen docking, CDR generation, and side-chain optimization through iterative refinement. By refining VHH–antigen complexes and redesigning CDR sequences, NanoDesigner addresses the interdependency between CDR conformations and antigen-binding geometry. Its workflow incorporates IgFold [118], HDOCK [104], Rosetta [119], FoldX [120], DockQ [121] and dr-sasa [122], together with DL-based strategies for CDR design and complex refinement [20,123,124]. This framework enables optimization of existing VHH–antigen complexes and exploration of alternative CDR conformations. However, its external dependencies and iterative refinement steps can increase computational demands.

Scaffold-based approaches also differ in their level of VHH-specific evidence. NanoDesigner [117] was developed and computationally benchmarked specifically for VHH design, whereas AiCDR [125] additionally generated VHH candidates with experimentally confirmed in vitro neutralizing activity. By contrast, DiffAb [20], dyMEAN [124] and ADesigner [123] provide Ab-specific sequence–structure design strategies by modeling antigen-conditioned CDR configurations. AntiFold [126], AntiBMPNN [127], and RosettaAntibodyDesign (RAbD) [128] were also developed primarily for conventional Abs, whereas ProteinMPNN [116] is a general protein-design model. These methods may provide useful components for VHH engineering, but their transferability requires VHH-specific assessment of framework integrity, CDR3 conformation, antigen contacts, and functional activity.

Future VHH design will likely combine de novo backbone generation with scaffold-based optimization. Generative approaches can explore new antigen-compatible structures, whereas scaffold-based methods retain the advantages of experimentally characterized VHH frameworks. Combining these strategies with improved structural prediction and experimental validation may provide a practical path toward developing functional pathogen-targeting VHHs.

## 6. Sequence-Based Deep Learning Models and an Illustrative VHH Representation Analysis

**Figure 5:**
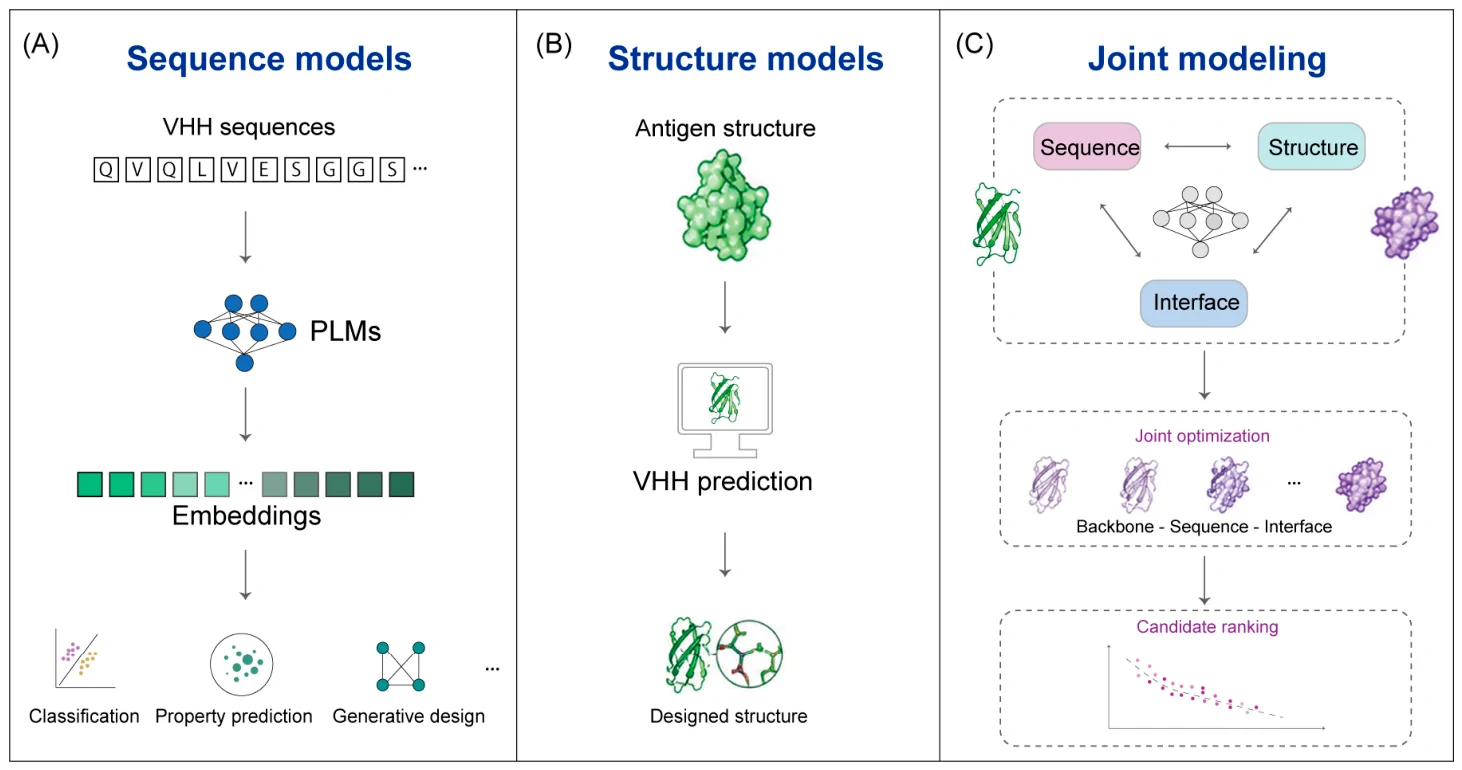
DL strategies for VHH design. (**A**) Sequence-based models use PLMs for VHH sequence representation, classification, property prediction and generative design. (**B**) Structure-based models, such as AlphaFold 3, predict VHH structures and VHH–antigen complexes for structure-guided design. (**C**) Joint sequence–structure models integrate sequence, structure and interface information to optimize VHH backbones, sequences and binding interfaces, followed by candidate ranking. Protein structures are schematic and do not represent experimental structures or model outputs.

Deep learning provides a flexible framework for learning representations directly from protein sequences, structures, and molecular interactions [95,129,130]. Because sequence variation, CDR conformation, and antigen recognition are closely coupled, current approaches can be broadly grouped into sequence-based, structure-based, and joint sequence–structure frameworks (Figure 5), which differ in the biological information they capture and the design tasks they support.

### 6.1. Sequence-Based Deep Learning Models for VHH Representation

PLMs have been increasingly applied in VHH engineering to provide sequence representations for classification, mutation-effect assessment, property prediction, and generative design [131,132,133]. In terms of model architecture, PLMs have progressed from general protein models to Ab- and VHH-specific ones (Table 5). The PLMs considered here also differ in their training domains. ESM-2 [134] and ProtBERT [135] are general protein models, whereas AbLang [136], AbLang-2 [137], AntiBERTy [138], AntiBERTa2 [139], IgLM [140], and ProGen2-OAS [141] were trained primarily on Ab repertoires. In contrast, nanoBERT [132] was trained specifically on VHH repertoires and has been evaluated computationally for VHH sequence modeling.

**Figure 6:**
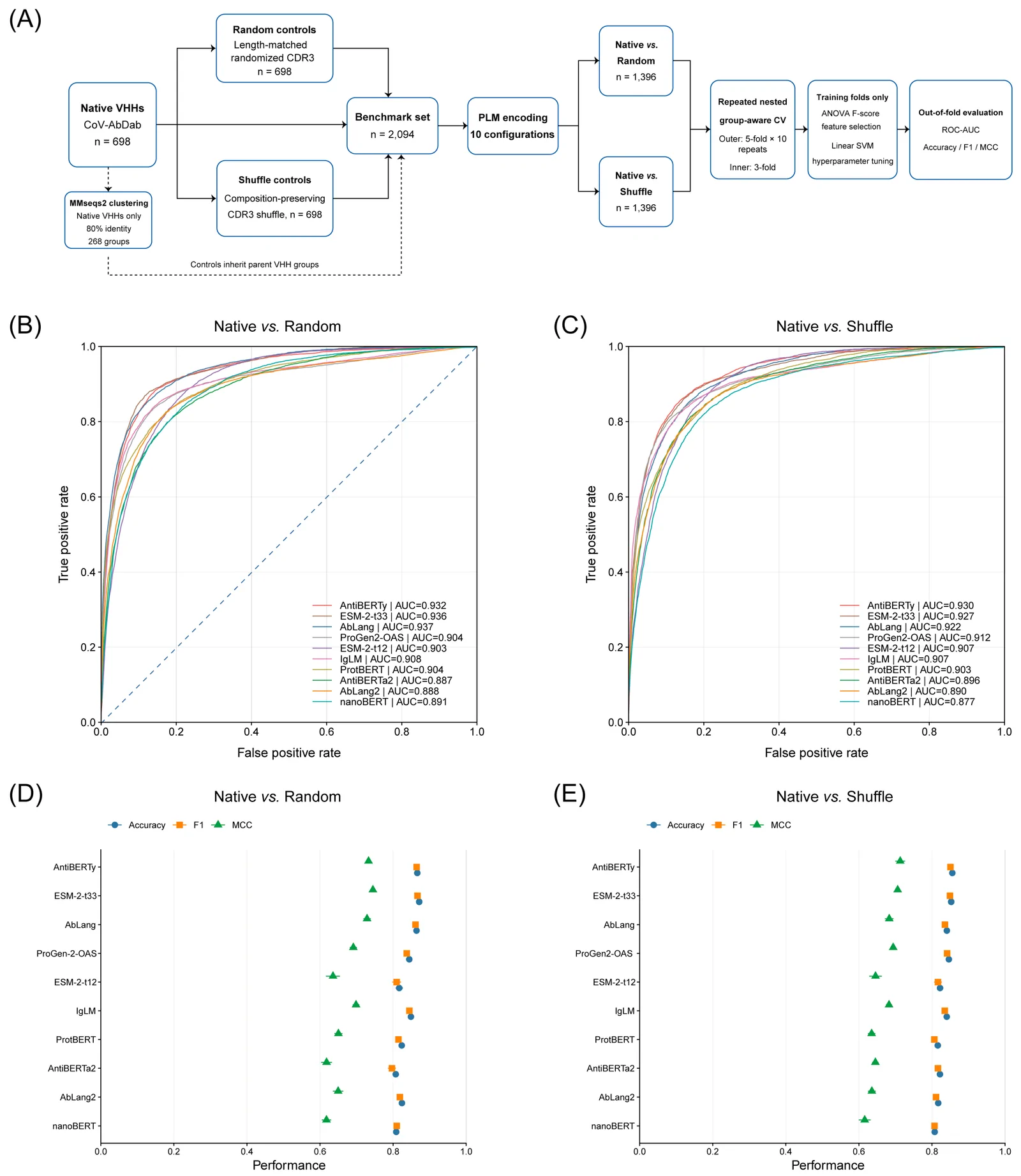
Illustrative task-specific comparison of PLM representations under controlled VHH CDR3 perturbation. (**A**) Analysis workflow. A total of 698 native VHH sequences from CoV-AbDab [39] were used to generate two matched control sets: length-matched randomized CDR3 sequences and composition-preserving CDR3-shuffled sequences (*n* = 698 each). Native VHHs were grouped by MMseqs2 (v18.8cc5c) [143] at 80% sequence identity, yielding 268 sequence groups; derived controls inherited the group assignment of their parent VHH. All 2094 sequences were encoded using 10 PLM configurations, including two ESM-2 variants, and evaluated in two independent tasks, native vs. random and native vs. shuffle. Feature selection and SVM hyperparameter optimization were performed exclusively within the training folds using three-fold group-aware inner cross-validation, while outer five-fold group-aware evaluation was repeated 10 times. (**B**,**C**) Mean ROC curves for the native vs. random (**B**) and native vs. shuffle (**C**) tasks, calculated from out-of-fold predictions across repeated five-fold evaluations. (**D**,**E**) Accuracy, F1 score, and Matthews correlation coefficient (MCC) for the native vs. random (**D**) and native vs. shuffle (**E**) tasks. Points indicate the mean across repeated evaluations, with error bars representing 95% confidence intervals.

Because PLMs differ in architecture, pretraining data, and learning objectives, as shown in Figure 6A, we compared ten representative PLM configurations using native VHHs and two controlled CDR3 perturbation sets under group-aware nested cross-validation. This analysis was designed to examine sequence representation rather than rank models for VHH engineering. In the native vs. random task, AbLang achieved the highest ROC-AUC (0.937), while ESM-2-t33 showed the highest accuracy, F1 score, and MCC (Figure 6B,D). In the native vs. shuffle task, AntiBERTy achieved the highest values for all four metrics, including a ROC-AUC of 0.930 (Figure 6C,E). The latter result suggests that the evaluated representations retain information sensitive to CDR3 residue order and context, but it does not reveal the biological basis of this distinction. The analysis also does not assess antigen recognition, binding affinity, biological activity, developability, or practical design performance. The results should therefore be interpreted only within these two sequence-discrimination tasks, consistent with the task-dependent performance reported by NbBench [142].

### 6.2. Structure-Based Deep Learning Models for VHH Design

While sequence-based PLMs provide useful sequence representations and capture statistical patterns in VHH sequences, they do not explicitly model the 3D geometry of antigen recognition [16,134,144]. Structural DL models have enabled the integration of protein conformations, residue-level geometry, and molecular interfaces into Ab and VHH engineering [87,118,145]. This is important for VHHs because antigen recognition depends on both CDR sequence and loop conformation, especially that of the extended CDR3 [14,146].

Structure-based VHH design has benefited from advances in protein structure prediction (Table 6). AlphaFold 3 [87] supported prediction of protein complexes, whereas IgFold [118] and NanoBodyBuilder2 (ImmuneBuilder) [145] model Ab variable domains and CDR conformations. These models provide structural hypotheses for interface analysis, affinity optimization, and computational VHH engineering.

Beyond structure prediction, DL models can generate and optimize VHH structures. RFdiffusion [115] generates protein backbones under predefined structural constraints. RFantibody [21] adapts this framework for epitope-conditioned generation of Ab and VHH binders. ProteinMPNN [116] can then design sequences compatible with generated backbones, whereas Ab-specific methods such as AntiFold [126] incorporate features of Ab variable regions.

Current workflows generally separate backbone generation, sequence optimization, and structural evaluation. Although effective, this modular strategy cannot fully capture the dependence between sequence and structure during antigen recognition. These limitations have motivated joint sequence–structure models that optimize both within a unified framework.

### 6.3. Joint Sequence–Structure Deep Learning Models for VHH Engineering

Sequence- and structure-based models provide complementary information for VHH engineering but remain limited when used independently. Sequence models capture evolutionary and contextual patterns without explicit geometry, whereas structure-based approaches typically treat structure prediction and structure-conditioned sequence design as separate tasks. Joint models can instead learn relationships among Ab sequences, conformations, and antigen interfaces.

As summarized in Table 7, sequence–structure learning has progressed from geometry-aware Ab refinement to antigen-conditioned and full-atom generation. Early graph-based models, including RefineGNN [147] and MEAN [148], jointly model CDR sequences and residue-level geometry, while HERN [18] incorporates antigen-interface information for paratope docking and Ab design. Subsequent models, such as DiffAb [20], dyMEAN [124], AbODE [149], AbDiffuser [150], and AbEgDiffuser [151], extend co-design to diffusion-based, antigen-conditioned, and full-atom generation. IgGM [152] further supports antigen-conditioned sequence–structure co-design of both conventional Abs and VHHs. Complementing these generative approaches, more recently developed NanoBind [153] can predict VHH–antigen binding, interface residues, and affinity ranges for candidate prioritization.

Despite these advances, VHH sequence–structure modeling remains constrained by data availability. Relatively few paired VHH–antigen examples are available, and only a subset includes experimentally resolved structures or quantitative affinity and functional measurements. Consequently, most generative models have been developed using conventional Ab data and evaluated mainly by sequence recovery, structural accuracy, or surrogate binding metrics. VHH-specific frameworks such as IgGM and NanoBind represent important progress, but their evidence remains largely computational and retrospective. Prospective validation is therefore needed to determine whether computational performance translates into antigen binding and biological activity, as discussed below.

## 7. Translational Challenges and Emerging Directions in AI-Guided VHH Engineering

**Figure 7:**
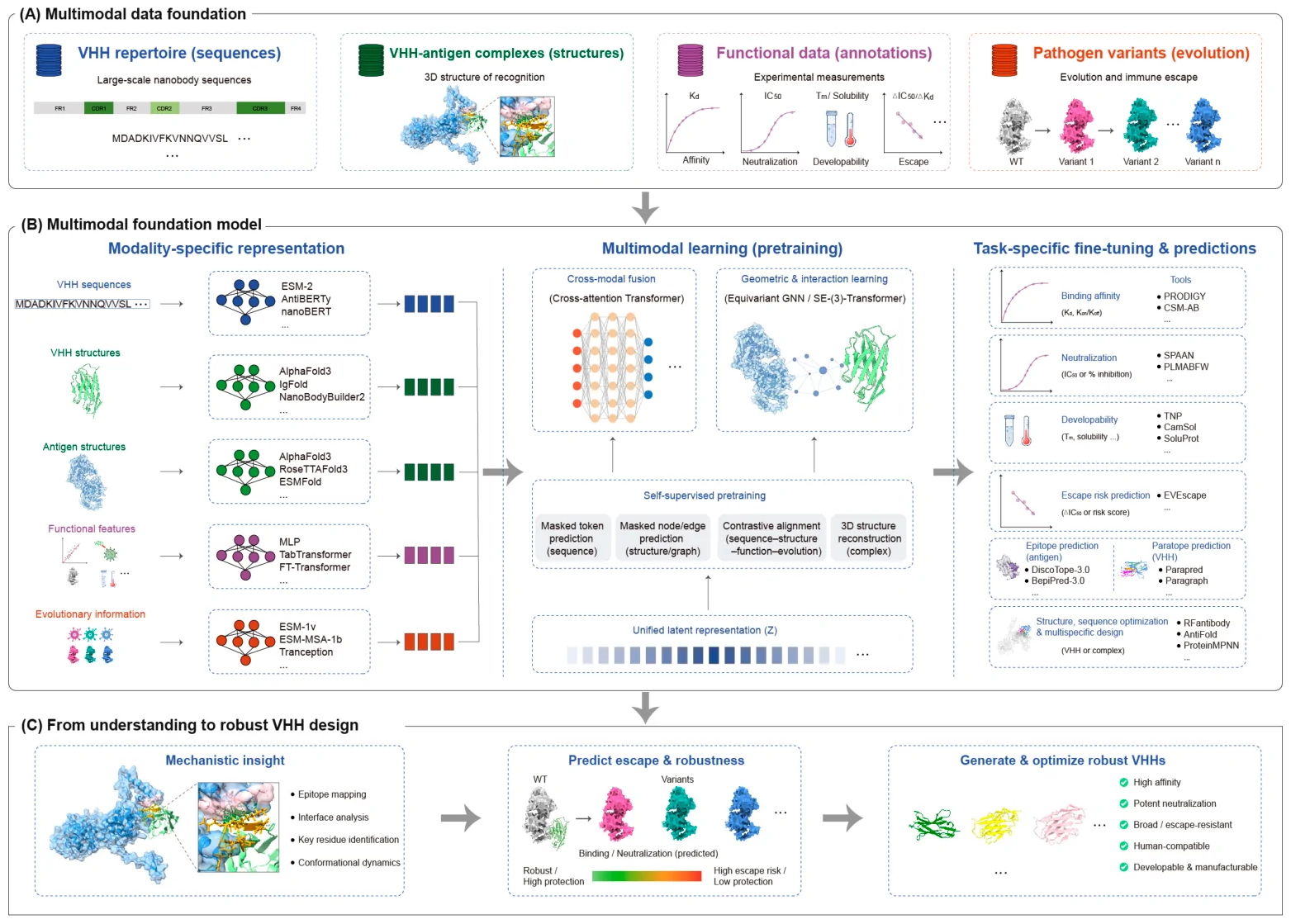
Conceptual framework for a pathogen-targeting VHH foundation model. (**A**) Large-scale VHH repertoires, VHH–antigen complex structures, functional measurements, and pathogen variants provide complementary data modalities for model development. Relevant VHH sequence, structure, affinity, and thermostability data are available from the resources summarized in Table 2, whereas pathogen variant and escape data require integration from additional sources. (**B**) Modality-specific representations could be learned using PLMs for VHH sequences, including ESM-2, AntiBERTy, and nanoBERT; structure predictors for VHHs and antigens, including AlphaFold 3, IgFold, NanoBodyBuilder2, RoseTTAFold, and ESMFold; neural networks for functional and production data, including multilayer perceptron (MLP), TabTransformer, and FT-Transformer; and evolutionary models, including ESM-1v, ESM-MSA-1b, and Tranception. Cross-modal attention and geometric learning could integrate these representations into a shared latent space. Task-specific fine-tuning could support affinity and neutralization prediction, developability and production assessment, escape-risk prediction, epitope–paratope mapping, humanization, multispecific design, and sequence–structure optimization. (**C**) Such a framework may improve mechanistic understanding of VHH–antigen recognition and support the design of functional, manufacturable, and escape-resilient VHHs. The schematic antigen structure was predicted using AlphaFold 3 from the rabies virus nucleoprotein sequence (GenBank accession NP_056793.1).

Experimental activity alone, however, does not ensure successful translation. Practical VHH development also requires manufacturability, compatibility with multispecific formats, humanization, and resilience to antigenic variation. Figure 7 outlines the multimodal data and modeling strategies needed to address these interconnected objectives.

### 7.1. AI-Guided Developability and Recombinant Production

Although DL approaches can integrate sequence, structure, and antigen context, most VHH design frameworks still focus on molecular plausibility or predicted binding. This leaves a gap between computational design and practical translation. A high-affinity VHH may still suffer from poor solubility, aggregation, low thermal stability, or inefficient recombinant expression, restricting formulation and scalable production [2,154]. Developability should therefore be considered during VHH design rather than only after candidate generation [155]. However, criteria established for conventional Abs may not transfer directly to VHHs. VHH-specific assessment should consider the exposed FR2 surface, hallmark residues, CDR3 length and conformation, and surface hydrophobicity and charge. The recently developed Therapeutic Nanobody Profiler (TNP) incorporates these features into an early-stage developability framework [156]. Nevertheless, computational profiling does not replace experimental developability testing.

Sequence-based models may help connect molecular design with production performance. DL has been used to predict heterologous protein expression in *Escherichia coli* and identify mutations that improve recombinant expression [157]. More recently, RP3Net combined protein- and host-derived representations to predict soluble recombinant protein production, with prospective validation on independent constructs [158]. Similar approaches could incorporate VHH sequence, stability, aggregation propensity, codon usage, signal peptides, host information, and expression yield. Because folding and secretion differ between *E. coli* and yeast, host-specific data will also be important [2]. Incorporating these production phenotypes into multimodal VHH models may help direct computational design toward manufacturable candidates (Figure 7B).

### 7.2. Multispecific and Combinatorial VHH Engineering

The modular architecture of VHHs facilitates their assembly into multivalent, bispecific, and multispecific formats. Combining VHHs that recognize different epitopes can increase avidity, broaden target coverage, and reduce dependence on a single antigenic site. Structure-guided multivalent VHHs against SARS-CoV-2, for example, showed enhanced neutralization and reduced mutational escape [8], while multidomain camelid Abs targeting influenza hemagglutinin achieved broad protection against influenza A and B viruses [159]. Bispecific VHHs targeting distinct regions of Ebola virus glycoprotein further illustrate the potential of complementary epitope recognition within a single construct [60].

However, combining two potent VHHs does not necessarily yield an optimal construct. Performance also depends on epitope compatibility, steric accessibility, domain orientation, and linker geometry. AI models could integrate structural, epitope, variant, and combination data to identify compatible VHH pairs and reduce empirical screening for multispecific design (Figure 7B).

### 7.3. Dynamic Recognition, Humanization, and Escape-Resistant VHH Design

Most computational workflows describe VHH–antigen recognition using static structures. This limitation is particularly important for VHH CDR3, which is often long and conformationally diverse; prediction accuracy generally decreases with increasing loop length [160]. AlphaFold and related structure predictors provide useful hypotheses but do not represent the full conformational ensemble of a flexible CDR3. Molecular dynamics simulations can complement structure prediction by examining alternative conformations and interface stability and have already been combined with machine learning for VHH affinity optimization [161]. However, their results depend on adequate sampling and require experimental validation. Therapeutic development adds a further constraint because camelid VHHs may require humanization. Framework substitutions can alter stability, CDR conformation, and antigen binding [30]. The sequence-based model AbNatiV has been applied to VHH humanization while preserving binding and stability [162]. Its successor, AbNatiV2, further improved VHH nativeness assessment using expanded camelid repertoire data [163]. These examples indicate that structural dynamics and human compatibility should be considered alongside affinity rather than treated as separate late-stage corrections.

Pathogen evolution adds another layer of uncertainty. Escape mutations can weaken potent VHHs when recognition depends on variable surface residues, whereas conserved or structurally constrained epitopes may be less tolerant of sequence change. The compact VHH architecture and extended CDR3 may offer an advantage by allowing access to recessed or cryptic epitopes that are difficult to reach with conventional Abs. AI models could combine antigen conservation, structural constraints, mutational data, and VHH–antigen interactions to identify epitopes with low mutational tolerance. Multispecific recognition could further distribute selective pressure across several sites, as demonstrated by multivalent VHHs that suppress SARS-CoV-2 escape [8]. The goal should therefore be to develop VHHs that retain activity across pathogen variants rather than merely bind a reference antigen with high affinity (Figure 7C).

## 8. Conclusions and Perspectives

VHHs are versatile molecular-recognition tools because of their compact architecture, structural stability, and ability to access sterically restricted epitopes. Their development is expanding from empirical screening toward structure-guided and AI-assisted engineering. This review frames pathogen-targeting VHH engineering along an application-guided structure–function–translation continuum. Within this framework, pathogen biology and functional epitope prioritization guide sequence–structure design, while developability, production, humanization, multispecificity, structural dynamics, and antigenic variation shape subsequent translation.

Current computational capabilities remain fragmented. Sequence models, structure predictors, and generative methods address different stages of VHH discovery, but most were developed using conventional Ab or general protein data and have limited VHH-specific validation. Moreover, diagnostic or therapeutic performance cannot be inferred from predicted binding alone. Functional activity, stability, manufacturability, human compatibility, and resilience to antigenic variation must therefore be treated as interconnected design objectives.

Multimodal foundation models may eventually connect these objectives, but their development remains a longer-term goal (Figure 7). Available datasets are fragmented and rarely link VHH sequence and structure with functional activity, production traits, and performance across pathogen variants. Progress will require standardized functional datasets and prospective experimental testing of model-derived candidates. Only then can multimodal models be meaningfully evaluated for multispecific design, humanization, manufacturability, and resilience to antigenic escape. Establishing these datasets and iterative computational–experimental workflows is therefore more pressing than increasing model complexity alone.

## Figures and Tables

**Table 1:** Recent reviews of computational and AI-assisted VHH engineering.

Review	Primary Scope	Representative Content	Organizing Perspective
Liu et al. [23]	VHH engineering and AI optimization	Libraries, display and production; NanoNet, NbBuilder2, and NbX	Engineering pipeline
El Salamouni et al. [22]	Computational modeling and design	Complex prediction, molecular dynamics, DSMBind, and Quenchbodies	Structure and dynamics
Reddy et al. [24]	Conventional, in silico, and machine learning approaches	Epitope prediction, docking, molecular dynamics, and ML-based generation	Discovery workflow
Zhu et al. [25]	Multiepitope and AI-assisted design	Viral multiepitope VHHs, HuDiff-Nb, RFdiffusion, and IgGM	Multiepitope engineering and humanization
Present review	AI engineering of pathogen-targeting VHHs	Functional epitope prioritization, design case of rabies virus, PLM benchmarking, and multimodal modeling	Structure–function–translation

**Table 2:** Public databases containing VHH and related sdAb data (accessed on 9 July 2026).

Name	Data Scope	Data Origin	Link	Ref.
INDI2	VHH sequence, structure, and metadata	Public repositories; computational integration	https://naturalantibody.com/indi2/	[34]
NanoLAS 1.0	VHH sequence, structure, and annotation	Multiple public sources; computational integration	https://oldversion.nanolas2.online	[35]
NanoLAS 2.0	VHH data with visualization and docking tools	Multiple public sources; computational integration	https://www.nanolas2.online	[36]
iCAN	VHH sequence, structure, and antigen data	Literature and PDB; curation	http://www.ican-nanobody.cn	[37]
Thera-SAbDab	Therapeutic Ab and sdAb data	Therapeutic records and PDB; curation	https://opig.stats.ox.ac.uk/webapps/sabdab-sabpred/therasabdab/search	[38]
CoV-AbDab	Coronavirus-binding Ab and sdAb data	Literature, patents, and PDB; curation	https://opig.stats.ox.ac.uk/webapps/covabdab	[39]
NanobodyThermo	VHH sequence and melting temperature data	Literature-derived experimental measurements	https://valdes-tresanco-ms.github.io/NbThermo/	[40]
ANDD	Ab/VHH sequence, structure, antigen, and affinity data	Multiple public datasets; computational integration	https://zenodo.org/records/18151718	[41]
sdAb-DB	Camelid VHH sequence	NCBI, PDB, and literature; curation	https://www.sdab-db.ca	[42]
PLAbDab-nano	VHH and VNAR sequence	Patents, literature, and public repositories; automated aggregation	https://opig.stats.ox.ac.uk/webapps/plabdab-nano	[43]
SAbDab-nano	VHH structure	PDB; curated experimental structures	https://opig.stats.ox.ac.uk/webapps/sabdab-sabpred/sabdab/nano	[44]
SAbDab2	Ab and VHH structure	PDB; curated experimental structures	https://sabdab.opig.stats.ox.ac.uk	[45]
SNAC-DB	Ab–antigen and VHH–antigen complex	PDB; computational curation	https://zenodo.org/records/20500158	[46]
PDB	Macromolecular structure	Primary experimental depositions	https://www.rcsb.org	[47]

Abbreviations: Ab, antibody; NCBI, National Center for Biotechnology Information; PDB, Protein Data Bank; sdAb, single-domain antibody; VHH, variable domain of the camelid heavy-chain-only antibody; VNAR, variable domain of the immunoglobulin new antigen receptor.

**Table 3:** Candidate antigen classes and priority regions for computational VHH design across pathogen types.

Pathogen	Antigen Class	Priority Antigen Regions	Representative Antigens
Viruses	Surface glycoproteins and fusion proteins	Receptor-binding sites; fusion regions; conserved exposed epitopes	SARS-CoV-2 Spike [57]; Influenza HA [58]; HIV-1 Env [59]; Ebola GP [60]
Viruses	Nucleoproteins and internal structural proteins	RNA-binding grooves; oligomerization interfaces; conserved exposed regions	Influenza NP [61]; Ebola NP [62]
Viruses	Viral enzymes and replication proteins	Catalytic pockets; cofactor-binding sites; replication interfaces	SARS-CoV-2 Mpro [63]; Influenza polymerase [64]
Bacteria	Adhesins and invasion factors	Receptor-binding domains; adhesive tips; exposed or repetitive surface epitopes	*E. coli* FimH [65]; *E. coli* F4 fimbriae [66]; *S. aureus* ClfA [67]
Bacteria	Toxins and secreted virulence factors	Receptor-binding domains; catalytic sites; translocation regions	Botulinum neurotoxin [68]; Anthrax PA [69]; Shiga toxin [70]
Bacteria	Outer-membrane, cell-wall, or envelope- associated proteins	Surface-exposed loops; conserved extracellular domains; uptake interfaces	*B. burgdorferi* OspA [71]; *E. coli* OmpA [72]
Parasites	Invasion and attachment ligands	Host-binding sites; invasion interfaces; adhesive domains	*P. falciparum* PCRCR complex (PfCSS/PfPTRAMP) [73]
Parasites	Surface-associated and transmission-stage antigens	Conserved exposed regions; protein-protein interfaces; surface epitopes	*T. brucei* VSG [74]; *P. falciparum* Pf12/Pf41 [75]; *P. falciparum* Pfs230 [76]
Parasites	Secreted or digestive enzymes and virulence-associated proteins	Catalytic sites; substrate pockets; host-interaction surfaces	*Leishmania* GP63 [77]; *S. mansoni* cathepsin D1 [78]; *F. hepatica* ES antigen [79]

**Table 5:** Representative protein, Ab, and VHH language models and evidence for VHH use.

Name	Scope and Training Basis	Architecture	Primary Role	VHH Evidence	Ref.
ESM-2	Protein; UR50/D	Transformer encoder	Sequence representation	None originally; Figure 6 analysis	[134]
ProtBERT	Protein; UniRef100	BERT encoder	Sequence representation	None originally; Figure 6 analysis	[135]
AbLang	Ab; OAS	Transformer encoder	Sequence completion and embedding	None originally; Figure 6 analysis	[136]
AbLang-2	Ab; OAS	Transformer encoder	Non-germline residue prediction	None originally; Figure 6 analysis	[137]
AntiBERTy	Ab; 558M Ab sequences	BERT encoder	Sequence representation and PLL scoring	None originally; Figure 6 analysis	[138]
AntiBERTa2	Ab; large Ab repertoires	RoFormer encoder	Sequence representation and residue prediction	None originally; Figure 6 analysis	[139]
IgLM	Ab; OAS	GPT-2 causal Transformer	Sequence generation and infilling	Computational nanobody generation; Figure 6 analysis	[140]
ProGen2-OAS	Ab; OAS	Causal Transformer	Sequence generation	None originally; Figure 6 analysis	[141]
nanoBERT	VHH; INDI sequences	BERT encoder	VHH representation and residue prediction	VHH-specific computational benchmark; Figure 6 analysis	[132]

Abbreviations: Ab, conventional antibody; INDI, integrated database of nanobodies for immunoinformatics; PLL, pseudo-log-likelihood; OAS, observed antibody space; VHH, variable domain of camelid heavy-chain-only antibody. “Figure 6 analysis only” indicates that no VHH-specific evaluation was reported in the original model study. The analysis in Figure 6 evaluates discrimination between native and artificially perturbed VHH sequences and does not validate antigen binding, biological function, developability, or engineering performance. None of the evidence presented here constitutes experimental validation of VHH performance.

**Table 6:** Representative structure-based models and current evidence for VHH use.

Framework	Original Scope	Primary Role	VHH Evidence	Ref.
AlphaFold 2/3	General biomolecules	Complex prediction	Independent computational benchmark	[86,87]
IgFold	Ab	Structure prediction	Direct computational benchmark	[118]
NanoBodyBuilder2	VHH	Structure prediction	Direct computational benchmark	[145]
RFdiffusion	General protein	Backbone generation	Experimental workflow validation	[21,115]
ProteinMPNN	General protein	Sequence design	Experimental workflow use	[115,116]
AntiFold	Ab	Inverse folding	Independent computational benchmark	[126]

**Table 7:** Representative joint sequence–structure learning models and evidence for Ab and VHH design and evaluation.

Model	Original Scope	Design or Evaluation Task	VHH Evidence	Ref.
RefineGNN	Ab	CDR sequence–structure co-design	No VHH evaluation	[147]
MEAN	Ab	Antigen-conditioned CDR design	No VHH evaluation	[148]
HERN	Ab	Paratope docking and design	No VHH evaluation	[18]
DiffAb	Ab	CDR sequence–structure co-design	No VHH evaluation	[20]
dyMEAN	Ab	Full-atom design	No VHH evaluation	[124]
AbODE	Ab	Antigen-conditioned CDR co-design	No VHH evaluation	[149]
AbDiffuser	Ab	Full-atom generation	Ab validation only	[150]
AbEgDiffuser	Ab	Antigen-guided co-design	No VHH evaluation	[151]
IgGM	Ab and VHH	Sequence–structure co-design	Direct computational benchmark	[152]
NanoBind	VHH	Binding, interface, and affinity prediction	Direct computational benchmark	[153]

## Data Availability

The data supporting the findings of this study are available from Rui-Si Hu upon reasonable request.

## Abbreviations

The following abbreviations are used in this manuscript:

3D	Three-dimensional
Ab	Antibody
ADCC	Antibody-dependent cellular cytotoxicity
AI	Artificial intelligence
cDNA	Complementary DNA
CDR	Complementarity-determining region
DL	Deep learning
ELISA	Enzyme-linked immunosorbent assay
F1 score	Harmonic mean of precision and recall
Fc	Fragment crystallizable
FR	Framework region
GNN	Graph neural network
HCAb	Heavy-chain-only antibody
IgG	Immunoglobulin G
IgNAR	Immunoglobulin new antigen receptor
IMGT	The international ImMunoGeneTics information system
INDI	Integrated database of nanobodies for immunoinformatics
ipTM	Interface predicted template modeling score
kDa	Kilodalton
MCC	Matthews correlation coefficient
MLP	Multilayer perceptron
Nb	Nanobody
NGS	Next-generation sequencing
OAS	Observed antibody space
PAE	Predicted aligned error
PBMC	Peripheral blood mononuclear cell
PCR	Polymerase chain reaction
PDB	Protein Data Bank
PLL	Pseudo-log-likelihood
PLM	Protein language model
PTM	Post-translational modification
RMSD	Root-mean-square deviation
RNN	Recurrent neural network
ROC-AUC	Receiver operating characteristic area under the curve
SASA	Solvent-accessible surface area
scFv	Single-chain fragment variable
sdAb	Single-domain antibody
SVM	Support vector machine
VH	Variable domain of the immunoglobulin heavy chain
VHH	Variable domain of the camelid heavy-chain-only antibody
VL	Variable domain of the immunoglobulin light chain
VNAR	Variable domain of the immunoglobulin new antigen receptor
